# Responsiveness of health care services towards the elderly in Tanzania: does health insurance make a difference? A cross-sectional study

**DOI:** 10.1186/s12939-020-01270-9

**Published:** 2020-10-12

**Authors:** Paul Joseph Amani, Malale Tungu, Anna-Karin Hurtig, Angwara Denis Kiwara, Gasto Frumence, Miguel San Sebastián

**Affiliations:** 1 Department of Health Systems Management, School of Public Administration and Management, Mzumbe University, Morogoro, Tanzania; 2 Epidemiology and Global Health, Umeå International School of Public Health, Umeå University, Umeå, Sweden; 3 Department of Development Studies, School of Public Health and Social Sciences, Muhimbili University of Health and Allied Sciences, Dar es Salaam, Tanzania

**Keywords:** Health insurance, Responsiveness, Elderly, Tanzania

## Abstract

**Background:**

Responsiveness has become an important health system performance indicator in evaluating the ability of health care systems to meet patients’ expectations. However, its measurement in sub-Saharan Africa remains scarce. This study aimed to assess the responsiveness of the health care services among the insured and non-insured elderly in Tanzania and to explore the association of health insurance (HI) with responsiveness in this population.

**Methods:**

A community-based cross-sectional study was conducted in 2017 where a pre-tested household survey, administered to the elderly (60 + years) living in Igunga and Nzega districts, was applied. Participants with and without health insurance who attended outpatient and inpatient health care services in the past three and 12 months were selected. Responsiveness was measured based on the short version of the World Health Organization (WHO) multi-country responsiveness survey study, which included the dimensions of quality of basic amenities, choice, confidentiality, autonomy, communication and prompt attention. Quantile regression was used to assess the specific association of the responsiveness index with health insurance adjusted for sociodemographic factors.

**Results:**

A total of 1453 and 744 elderly, of whom 50.1 and 63% had health insurance, used outpatient and inpatient health services, respectively. All domains were rated relatively highly but the uninsured elderly reported better responsiveness in all domains of outpatient and inpatient care. Waiting time was the dimension that performed worst. Possession of health insurance was negatively associated with responsiveness in outpatient (− 1; 95% CI: − 1.45, − 0.45) and inpatient (− 2; 95% CI: − 2.69, − 1.30) care.

**Conclusion:**

The uninsured elderly reported better responsiveness than the insured elderly in both outpatient and inpatient care. Special attention should be paid to those dimensions, like waiting time, which ranked poorly. Further research is necessary to reveal the reasons for the lower responsiveness noted among insured elderly. A continuous monitoring of health care system responsiveness is recommended.

## Background

In low and middle-income countries (LMIC), health care systems are likely to be challenged by the rapidly increasing numbers of the elderly population [1–5]. A large proportion of this group are socio-economically disadvantaged and live in rural areas with poor health care infrastructure [6, 7]. Compounding this situation is the increasing need for health care services adapted to non-communicable diseases like diabetes, hypertension, a variety of cancers and deteriorating physical mobility, which predominantly affect the elderly [8, 9]. Many LMICs have initiated reforms to their health care systems with a focus on improving the availability and accessibility of health care services for this vulnerable population group. These reforms are in line with the World Health Organization’s (WHO) proclamation concerning universal health coverage (UHC), which focuses on building an enabling health system that is able to provide equitable health care access and financial protection to people, regardless of their capacity to pay [10]. This milestone requires a political commitment and acceptability, particularly in sub-Saharan countries, where health systems are generally weak [4].

When countries decide to reform their health care systems, monitoring and evaluation become an inescapable strategy for ensuring good performance [11]. In 2000, the WHO emphasised the need to put mechanisms in place to ensure the health system’s ability to improve the health of the population, to protect the poor from potential care expenditures and to respond to legitimate expectations of people, thereby increasing the degree of responsiveness [12].

The concept of responsiveness was therefore introduced to capture patients’ experience with the health system based on a common set of non-health domains [11–15]. These include the quality of basic amenities, choice, confidentiality, autonomy, communication and prompt attention. As they are developed from an extensive array of disciplines, responsiveness domains analyse the function of the health care system from the way patients experience care, the treatment procedures and the environment around the services [16–18]. Although responsiveness has increasingly been promoted as a key goal of any health system, its measurement remains scarce [11, 12, 17, 19, 20]. Studies on responsiveness have been more common in high-income countries [14, 21–23] than in LMICs [17, 24]. In the former, health care users have mainly reported concerns regarding trust, long waiting times, lack of empathy and friendliness, and limited involvement in decision-making [22, 23, 25]. In the case of the LMICs, different studies [9, 13, 15, 20] have shown choice of service provider, autonomy, prompt attention, quality of basic amenities and confidentiality as important areas of concern in terms of responsiveness. In sub-Saharan Africa, studies from Nigeria [11] and South Africa [26] have also shown the usefulness of the responsiveness domains in examining the operationalisation of health systems in the context of health insurance schemes. These studies identified the domains of access, autonomy, communication and prompt attention as important areas that the management of health insurance (HI) should work on in order to improve the responsiveness noted among the insured.

Towards the end of the 1980s, Tanzania, like other LMICs, was compelled to improve its health care system through attempts to minimise budgetary constraints [27]. These improvements consisted of the introduction of HI to the country as part of the primary health care strategy.

Community Health Fund (CHF) was piloted in the Igunga district in 1996 and was later introduced in other districts across the country as a voluntary scheme for rural households and their dependents, who agreed to contribute the same amount of premium. Although National Health Insurance Fund (NHIF) was originally introduced in 2001 as a mandatory scheme to cover public servants, currently its coverage has been extended to the informal sector as well. Both schemes strive to improve access and utilisation of basic health care services by the poor and the vulnerable population, including the elderly, with the goal of achieving UHC [28–30]. Research has shown that HI can contribute to improving the health care system’s ability to deliver health services, particularly among low socio-economic groups [11, 31]. However, little is known about how HI contributes to the responsiveness of health care services. To our knowledge, only one recent study has addressed the issue of responsiveness of primary health care services in Tanzania [32], but none has focused on the role of HI or elderly care.

Thus, this study aimed to assess the responsiveness of the health care services among the insured and non-insured elderly in Tanzania and to explore the association of HI with responsiveness in this population, in order to contribute with relevant knowledge to improve the performance of the health care system for the elderly in the country.

## Methods

### Study setting

The study was conducted in Nzega and Igunga districts in the Tabora region, which is located in western-central Tanzania. According to the 2012 census, the region had 2.3 million inhabitants, of which 901,979 resided in Nzega and Igunga districts [33]. The number of people aged 60 years and above living in these districts was 50,547, approximately 5% of the total population. We chose the two districts for logistical reasons, as the two are neighbours, both with a majority of the elderly residing in rural areas, and Igunga was the first district in the country to experience the CHF. In both districts, primary and secondary health facilities are available and offer health care services to the elderly regardless of their insurance status. While the retired elderly from the public sector who had already joined the NHIF are covered until their death, those who are not can voluntarily join the CHF. An insured elderly person is entitled to a broad range of free services, including outpatient consultation, prescriptions, surgical services, inpatient care services, physiotherapy and rehabilitation services, optical and dental health services [28, 34, 35].

### Sample size and sampling procedures

This study is part of a broader project assessing the role of HI among the rural elderly. A household-based survey of elderly people aged 60 years or more, living in Igunga and Nzega districts, was conducted between July and September 2017. A multistage sampling technique to select the wards and villages in each district was applied. First, through a convenient sampling technique, fourteen wards were selected randomly, seven from each district (around 1/5 of the total number of wards from each district) based on population size and logistics. Second, a total of 58 villages that were geographically reachable from the fourteen wards were randomly selected by using a lottery method. Third, hamlet officers helped us to identify and select 25 to 44 households with an elderly person from each village, depending on village size. Lastly, one respondent, either male or female, was randomly selected and interviewed from each household. The inclusion criteria for respondents were: to be aged 60 years or over; currently living in the selected districts; and visiting an outpatient or inpatient service in the last three or 12 months. Given the lack of studies on responsiveness in Tanzania and the variety of results found in the literature, we based our outcome prevalence on one of the studies with the lowest overall responsiveness score [13]. Based on a 40% prevalence of good responsiveness in outpatient care, a design effect of two, a 95% confidence interval and power of 80%, 733 participants were determined to form the sample size. This sample was used to obtain a representative group of males and females separately.

### Data collection

A pre-tested household survey was first applied to understand the perception of the insured and uninsured elderly with regard to outpatient and inpatient health care services received in the past three and 12 months, respectively. We employed eight data collectors who were fluent in the Swahili and Sukuma languages and had at least a bachelor’s degree in social sciences. Before starting data collection, the research assistants received training and became accustomed to the questions in order to reduce misunderstandings of the domain terms by themselves or the respondents.

### Defining the variables

The responsiveness questions were based on the short version of the WHO multi-country survey study [14]. The responsiveness domains were measured by using the five ordered Likert scale options: 1 = very good, 2 = good, 3 = moderate, 4 = bad and 5 = very bad. The general question addressing the six domains was: ‘For your most recent visit to a health care provider/overnight stay, how would you rate the following: i) cleanliness of the facility’s inside environment; ii) freedom to choose health care provider; iii) freedom to talk privately to the provider; iv) involvement in deciding treatment; v) clarity of explanation by providers, and vi) time waited before being attended’. Cronbach’s alpha was used to measure the reliability of the instrument, which ranged, depending on the domain, between 0.68–0.89, which can be considered as acceptable.

Health insurance status was determined with a ‘Yes/ No’ question by asking the elderly if they possessed HI (public or private). The elderly were requested to show their HI membership cards (all did), as well as to state the date they joined the scheme.

The sociodemographic factors included were: i) sex/gender, identified as male or female; ii) age, categorised as between 60 and 69, 70–79 and > 79 years old; iii) marital status was divided into: married (currently married and cohabiting) and other (widows, separated and never married); iv) education was categorised as none, low education – those with a primary education or less – and high education, those with a secondary education or higher; and v) income was determined by asking about the total income of the individual elderly and categorised as less or equal to $22.50 and above $22.50. The cut-off is based on the Tanzanian 2011–2012 basic need poverty line estimates, which stood at 236,482Tsh (approximate to $22.5) per adult per month in 2018.

### Ethical clearance

The Research and Ethics Committee of Muhimbili University of Health Sciences reviewed and approved the study protocol in May 2017 (reference number 2017-05-24/AEC/Vol.XII/70). Permission to conduct data collection from the District Executive Directors of Igunga and Nzega districts was obtained. Then, an informed written consent was obtained from participants and verbal consent was obtained from those who could not read and write, after the local guide had introduced the research assistant and the procedures for research in each household including their rights to participate or withdraw from the study.

### Data analysis

The data were entered into Epi Info and analysed with STATA version 15. First, a descriptive analysis presenting the characteristics of the study sample was carried out. Then, the degree of responsiveness by type of care based on the five categories of responses (1 = very good to 5 = very bad) was analysed. To obtain a responsiveness index, the scores for each domain were first reverse coded to 5 = very good and 1 = very bad and then added, resulting in an index ranging from six, indicating the lowest, to 30, the highest score [2]. Chi-square tests were applied to compare the health systems’ responsiveness domains according to the possession or not of HI. Since a non-normal distribution of the index was observed, a median quantile regression (50th percentile) was used to explore the specific association of the responsiveness index with HI ownership and sociodemographic factors. Statistically significant variables (*p*-value < 0.05) in the crude model were included in the adjusted model.

## Results

### Characteristics of the respondents

Table 1 portrays the descriptive characteristics of the elderly people who were involved in this study. The final sample included 1453 and 744 elderly people who reported using outpatient and inpatient services in the last three and 12 months, respectively. A similar distribution of respondents between outpatient and inpatient care was observed for the different sociodemographic variables. Study participants were mostly younger (60–65 years), not currently married, with no education and low income. While half of the respondents in the outpatient group were insured, the coverage increased to 63% in the inpatient group.

**Table 1:** Characteristics of respondents by use of health care services

**Characteristics**	**Outpatient** **(*n*** **= 1453)**	**Inpatient** **(*n*** **= 744)**
**Gender**		
Male	704 (48.45%)	349 (46.91%)
Female	749 (51.55%)	395 (53.09%)
**Age (years)**		
60–69	849 (58.43%)	424 (56.99%)
70–79	378 (26.12%)	189 (25.40%)
> 79	226 (15.55%)	131 (17.61%)
**Marital status**		
Married	640 (44.05%)	344 (46.24%)
Other	813 (55.95%)	400 (53.76%)
**Education**		
None	828 (56.99%)	425 (57.12%)
Low	563 (38.74%)	289 (38.84%)
High	62 (4.27%)	30 (4.04%)
**Income**		
≤ 22.50$	970 (66.75%)	504 (67.74%)
> 22.50$	483 (33.24%)	240 (32.26%)
**Health insurance**		
No	722 (49.69%)	275 (36.96%)
Yes	731 (50.31%)	469 (63.04%)

### Performance of the responsiveness domains by health insurance

**Fig. 1:**
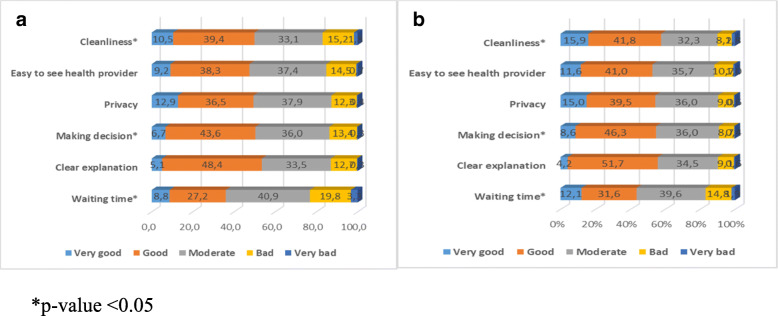
Percentage of participants rating the responsiveness domains in outpatient care by health insurance ownership. **a** Insured. **b** Uninsured

Through ratings, the experience of the elderly regarding the six responsiveness domains based on their insurance status was explored. In general, good (including very good, good and moderate) responsiveness was reported in all domains for outpatient care except waiting time. The uninsured elderly reported better responsiveness than the insured in all domains of outpatient care including cleanliness of the facility, involvement in treatment decisions and waiting time which were statistically significant (Fig. 1a and b).

**Fig. 2:**
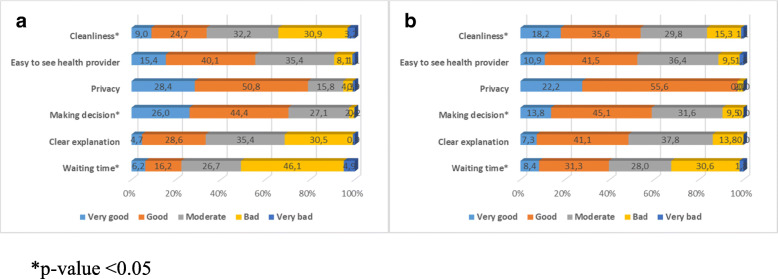
Percentage of participants rating the responsiveness domains in inpatient care by health insurance ownership. **a** Insured. **b** Uninsured

Similar to outpatient care, the uninsured elderly reported better responsiveness than the insured in all domains of inpatient care. The same dimensions as in outpatient care, cleanliness, making decisions and waiting time performed statistically lower among the insured compared to the uninsured (Fig. 2a and b).

### Regression analysis

Table 2 shows the results of the crude and adjusted regressions of the median quantile analyses estimating the association between HI and both the outpatient and inpatient overall responsiveness index, adjusted for sociodemographic variables.

**Table 2:** Results of crude and adjusted median quantile regression models for responsiveness to health care services

	**Outpatient (*n* = 1453)**		**Inpatient (*n* = 744)**
**Characteristics**	**Crude model**	**Adjusted model**	**Crude model**
**HI**			
No	Reference category		
Yes	−0.7 (−1.17, − 0.32)	− 1 (− 1.45, − 0.45)*	−1.8 (−2.36, − 1.24)*
**Age (years)**			
60–69	Reference category		
70–79	−0.6 (−1.10, − 0.10)	− 1(− 1.70, − 0.29)*	−0.5 (− 1.15, 0.17)
> 79	−1 (− 1.65, − 0.44)	−2 (−2.85, − 1.14)*	0.3 (− 0.44, 1.06)
**Gender**			
Male	Reference category		
Female	−0.7 (−1.12, − 0.27)	− 1 (− 1.60, − 0.39)*	−0.1 (− 0.63, 0.48)
**Marital status**			
Other	Reference category		
Married	−0.4 (−0.87, − 0.02)	−1 (−1.60, − 0.39)*	− 0.0 (− 0.57, 0.54)
**Education**			
None	Reference category		
Low	0.2 (−0.12, 0.70)	1 (0.49, 1.50)*	0.2 (−0.30,0.84)
High	1 (0.17, 2.30)	2 (0.78, 3.21)*	1.3 (−0.30, 2.70)
**Income**			
< 22$	Reference category		
> 22$	0. (−0.38, 0.51)		0.3 (−0.34, 0.84)

*p-value < 0.05

### Outpatient care

Results of the crude and adjusted regression models showed a negative statistical association between HI and responsiveness regarding outpatient care. The responsiveness perceived by the insured elderly was one unit less (− 1; 95% CI: − 1.45, − 0.45) than that of the uninsured elderly. In addition, a negative statistical association between age, gender and marital status with responsiveness was observed. The increase in age decreased the probability of reporting better responsiveness by one unit (− 1; 95% CI: − 1.70, − 0.29) among the group aged 70 to 79 years and two units (− 2; 95% CI: − 2.85, − 1.14) in the group aged 79 years or older, as well as among females and married people (− 1; 95% CI − 1.60, − 0.39), whereas high education (+ 2; 95% CI: 0.78, 3.21) and high income (+ 1; 95% CI: 0.36, 1.63) were associated with higher responsiveness.

### Inpatient care

The results of the crude models also showed a negative association between HI and responsiveness in relation to inpatient care (− 2; 95% CI: − 2.69, − 1.30). No adjusted models were conducted, since none of the sociodemographic variables (age, gender, marital status education and income) showed a significant association with responsiveness to inpatient care in the bivariate regression.

## Discussion

To our knowledge, this is the first study analysing the responsiveness of health care services in Tanzania by insurance status. In this section, we first discuss the performance of the different domains and then the difference in responsiveness perceived by the insured and the uninsured elderly.

### Responsiveness in outpatient and inpatient care

Based on our findings, both the insured and uninsured elderly reported good responsiveness (very good/good/moderate ≥50%) in all domains of outpatient and inpatient care. High scores in all domains were also found in the Tanzanian study that explored responsiveness in primary health care among the general population [32]. In our study, the perceived health care responsiveness was, however, lower among the insured compared to the uninsured elderly in all domains of both types of care. Our results are in line with the findings of similar studies from sub-Saharan Africa. In a study conducted in South Africa among insured and uninsured older adults (50 years and above), a good health system responsiveness was observed in all domains of outpatient and inpatient care [15]. Similar experiences have been reported by insured and uninsured patients in Nigeria, who indicated a high responsiveness in outpatient care [11].

Three domains – access (ease of seeing a health provider), confidentiality (privacy) and autonomy (involvement in decision-making) – performed better among both the insured and uninsured elderly in outpatient and inpatient care services. This finding differs from the results of the previous South African study [15], which reported patient dissatisfaction with the access and autonomy domains of the health care system. The observed better responsiveness concerning access shown in our study may be a result of the government’s ongoing efforts to improve service delivery, particularly at the primary health care level, which is widely available in rural areas. According to Röttger et al. [23], users of health care services expect a high level of privacy and assurance that whatever personal information they discuss with health care providers is safeguarded. In our study, the confidentiality domain performed satisfactorily, similar to the South African study [15] that reported high responsiveness (74.2%) in that domain. However, in our study setting, many health facilities were small, had limited space for patient–doctor meetings and used the available space for multiple activities. It could be that elderly patients were comfortable with the level of confidentiality because it had recently improved, and/or they did not have other experience to compare. Nevertheless, there is a need to readjust the facility’s space and remind health care providers of the ethics of information privacy. Autonomy describes the rights of a patient to medical information and to make informed choices [11]. Involving the elderly in making decisions about their health may enhance patient–doctor relationships, which are important in the care process [25]. Although information asymmetry is common in health care settings, the findings from our study appear to highlight an existing good relationship between health care providers and patients in the sense that it gives the patient a sense of control and responsibility and hence, allows them to be involved in the care activity [36].

Our results revealed a concern by the elderly regarding three responsiveness domains: prompt attention (waiting time), quality of basic amenities (cleanliness) and communication (clear explanations). These findings are similar to previous studies on health care responsiveness among older adults in South Africa [15], China [13] and Nigeria [11]. Nevertheless, our scores regarding prompt attention were extremely low (18.15% in outpatient and 21.85% in inpatient care) compared to those of South Africa (58.2% for outpatient and 68.6% for inpatient) and Nigeria (68% for outpatient care). In line with other research, dissatisfaction of the elderly may be associated with overcrowding, understaffing, limited geriatric skills, delays in reception, unavailability of recommended medicine, attitude of providers towards the elderly and processing insurance claims [11, 37, 38]. Similar to prompt attention, neither insured nor uninsured patients were satisfied with the cleanliness of the facilities. These findings are different from other studies [26, 37] in which this domain was scored highly and deemed important. In our study, cleanliness was perceived as poor (21.35%) for inpatient care compared to the South African study, which was 71.3% [26]. There is definitely a need for health care managers to improve the cleanliness of their facilities in order to offer a quality service. In line with the WHO [12], communication is also very important in improving the delivery and utilisation of health care. However, the dissatisfaction observed with communication in this study may imply that providers do not take enough time to listen to and understand the problems of elderly patients. This is a not a good practice, as it disempowers the service users, makes them feel uncomfortable with the provider and may lead to decreased trust in the health care delivery system.

### Factors associated with responsiveness

The elderly with HI reported worse responsiveness compared to the uninsured, in the adjusted quantile regression models. This finding can appear to be contradictory at first sight. Although research from Ghana has shown similar results [39], in which insured patients tended to perceive worse quality of health care, a study from Burkina Faso [40] showed no difference in the quality of health care among insured and uninsured patients. Two main reasons could be argued for the difference in our study: difference in procedures when visiting a health facility and unfulfilled expectations. In the Tanzanian health care setting, an insured elderly person has to go through a long process before being seen by a doctor. They start by submitting the insurance card at the reception and then wait while undergoing verification through the computer system, which may take a long time due to overcrowding. However, the uninsured pay cash and get the services immediately, which is a quicker process with commonly shorter queues than that of the insured patients. Furthermore, the fact that patients are given appointments for a particular day but not time, and may not be seen immediately due to the ‘first come-first-served’ modality, added to the overcrowding of health facilities particularly in the insured section, can contribute to this finding [37]. A similar experience from Ghana showed that dissatisfaction of the insured was associated with long waiting times, inadequate information regarding services, poor staff attitudes, non-observance of the queuing process and perceived low quality of drugs [39]. Related to the second explanation, insured patients may expect to be attended by professionals who show concern for and understanding of their health problems, to experience shorter waiting times and to receive better quality services than the uninsured. If this does not happen, responsiveness can be perceived as being worse.

Among the independent variables, older age, female and being married showed a negative statistically significant association to responsiveness in outpatient care. The result regarding age is, however, opposite to other studies [9, 15] that have reported more responsiveness by older people. One possible explanation might be that health care services are used more often with age, making elderly more negative towards them. Literature offers different findings regarding gender and responsiveness. In the South African study, female inpatients indicated higher health care responsiveness [15], whereas in studies from Ethiopia [41] and Ghana [9], gender differences did not influence the responsiveness perception among older patients. This difference might require further exploration. Higher educational attainment tended to be positively associated with perceived responsiveness in outpatient and inpatient care. This finding is similar to other studies [42–44] which showed increased responsiveness with higher education, but it differs from the findings of a study in Ethiopia [45]. A probable explanation might be that elderly people with higher education have a better knowledge of what services they need, as well as greater ability to interact with the providers and navigate within the system [19].

### Methodological considerations

The survey used to explore the responsiveness of health care services was based on the responsiveness questions included in the WHO multi-country responsiveness survey study [14], which allowed for consistency and comparison with other studies. The response rate was high (above 80%), probably due to the recruitment of research assistants who were fluent in the local language and the culture of the study respondents. The fact that our sample size was relatively high, with both males and females represented, increases the internal validity of our findings. However, generalisation of the results to other parts of the country should be undertaken cautiously. Several measures were taken to minimise the possibilities of bias and misinterpretation by both the interviewers and the respondents. In order to reduce interviewer misinterpretation and thus respondent bias, we conducted a pilot test of the instrument, with thorough training for the research assistants. The responsiveness questions related to health care utilisation might have created recall bias. This was partly dealt with by requesting to see HI cards and hospital registration numbers for a randomly selected number of respondents during interviews. Selection bias was partly taken into consideration because of the randomisation process of the participants’ selection. Finally, we could not distinguish to which kind of HI participants belonged, which could have influenced the perception of the responsiveness domains.

## Conclusion

To our knowledge, this is the first study analysing the responsiveness of the health care services in Tanzania with a focus on insurance status among the elderly. The uninsured elderly reported better responsiveness in all domains than the insured, and a negative association between HI and the responsiveness index in outpatient and inpatient care was observed. The results suggest that further attention to the HI procedure is needed in order to further improve the responsiveness of the health care services. For service providers, the results highlight the importance of considering needs, values and preferences of elderly patients to improve their experience and perceptions as well as to meet their expectations of the health care provided. Policymakers would need to take measures in order to improve three main aspects of care – communication between doctors and patients, prompt attention and cleanliness – to meet the expectations of elderly patients. The government of Tanzania is planning to improve in the nearest future access and to ensure UHC for all people. It would be worth undertaking careful monitoring of the process of implementation of these strategies from a responsiveness perspective.

## Data Availability

Not available.
